# Acknowledgement to reviewers

**DOI:** 10.1530/EO-3-A1

**Published:** 2023-01-25

The editorial board wishes to thank the following individuals who have helped to review manuscripts for *Endocrine Oncology* during 2022. Their assistance is gratefully acknowledged.

A Abazarikia

F Acconcia

B B Aggarwal

A Albage

D Albano

E K Alexander

M Allinovi

M Almeida

K Almstrup

A Al-Qasem

C Amaral

L Andereggen

D Andersen

H Arima

J Arnold

N Arslan

E Arthur

K Ashida

S Asioli

S P Balasubramanian

S E Baldeweg

S Balk

I Bancos

H R Baradaran

R Barroso-Sousa

S Basu

I M Bensenor

C L Bevan

K N Bhalla

S Bhattacharyya

N R Biermasz

J Bil

I Binse

C V Bishop

C S Blesson

C A Blum

M Bongiovanni

J Bornschein

C Bousquet

J Bowden

T Brandenburg

I Brownell

K L Burnstein

M E Cabanillas

D Campana

C Caputo

P Caron

L Carrillo

R T Casey

J P Castaño

E Castro

A C B Cato

P P Caturegli

V Cavailles

F Ceccato

J M Cerutti

D Chakravarti

J B Chambers

K K Chan

H Chen

S Chen

B-H Chew

H Choi

P Ciana

A Ciarrocchi

R Cioffi

M Cives

R Clarke

D Clement

P Collini

R Colomer

S Corbetta

R R Correa

J M Costa

B Cremer

H O D Critchley

J Crona

L L Cunha

R Dadu

P Dahia

A F Daly

M De Felici

W W de Herder

F H de Jong

P de Ronde

S M C De Sousa

M Debono

S M Dehm

E D Desouky

S S Dhage

G Di Dalmazi

A Di Marco

H J Ditzel

H-J Du

C Durante

G Eisenhofer

J Elhasnaoui

A Elias

E Elkord

D Ellison

C Eloy

G D Eslick

S Ezzat

A Fabisiewicz

J Faivre

T Falcone

O O D Faluyi

M Fassnacht

R A Feelders

F Feng

M Fenwick

F Filippi

L Filippi

L Fishbein

M C Fragoso

H Fukuoka

M R Gadelha

M D Gahete

M R Galdiero

D Gallo

J S Garcia

A Garrahy

M Garutti

J Geisler

E P Gelmann

H K Ghayee

D A Gibson

P Gimenez-Bonafe

O Gimm

L Giovanella

P Giovannelli

M Girotra

N J Gittoes

G Giuffrida

A Glover

G Grani

S Greenberg

S Grodski

A B Grossman

M Gurnell

A Hajj

T R Halfdanarson

P Hall

D M Halperin

O Hamidi

J W Hamner

H Hanieh

D C Hassane

P Haycock

A Hermawan

M C Hernández

S C Hewitt

C E Higham

L J Hofland

Y Horimoto

M C R Horta

Y-C Hsu

I T Huhtaniemi

A Ibáñez-Costa

W Inder

K Ito

R Jain

J A M J L Janssen

M Jordà

C C Juhlin

C K Jung

G Kaltsas

G A Kaltsas

E Kandil

N Karavitaki

E Kassi

E Kebebew

K W Kim

S-Y Kim

E T Kimura

K Kiseljak-Vassiliades

C M Kitahara

N Kiyota

F G Klinger

A König

S Koutros

S Krug

M Labrie

H Lahner

M Lambertini

B Lapauw

B Li

J Li

M Li

T-C Li

K E Lines

G Livera

S K Logan

C Lok

J Lopez-Mattei

I Lupi

R M Luque

Q Ma

R Ma

B Madeo

P Malandrino

M Mannelli

N Mansencal

A Maraschini

M Marazuela

G Marone

L J Martin

N Matikainen

K Matsumoto

I McEwan

M McIlroy

L McWilliams

T Mercantepe

O Mete

P Miccoli

J Miller

L Min

H Minami

G Mintziori

Z Mitri

N Mitsiades

M Miyashita

F Montemurro

M B Moravek

A Mousavi Khaneghah

J Munkley

P S Nelson

P J Newey

H Nishioka

B Nixon

I J Nixon

M Nomura

K E Oberg

G Occhi

K Oktay

H Olsen

F Orafidiya

A Orlandi

J H Ostrander

M Pajaro

J J Palvimo

F Pani

F Panzuto

M Papaleontiou

U-F Pape

T Pappa

A Pariente

S-H Park

S Partelli

S H S Pearce

V Pelekanou

G Pellegriti

P Pellikka

L G Perez-Rivas

A Perry

P Petrossians

F Pitoia

E J Podesta

T D Poeppel

F Ponti

A C Powers

A Prejbisz

A Prete

G W Randolph

G Raverot

M Razi

N S Reed

M M M Refaie

J K Richer

R P Riechelmann

G Riesco-Eizaguirre

G Rindi

S Robertson

M Robledo

V Rochira

R J Rodgers

F Roncaroli

C L Ronchi

A Rotte

M Rubio-Almanza

N Sachithanandan

M Sadar

S Saji

R Samji

J J Sancho-Insenser

N Sang-Yoon

H A Santos

H Sasano

S Saso

P T Saunders

P E Scherer

S Schonfeld

T Schuurman

D Scordamaglia

L A Selth

M Sherlock

F Shi

S B Sidhu

J A Simpson

R Sippel

S Sixou

P Soares

M Sobrinho-Simões

C Soekmadji

M Song

S Sorrenti

A Spanu

N Spears

J Spencer-Segal

C Spitzweg

R Steeds

M Stelmachowska-Banas

C E Stiles

K-H Storbeck

A Suzuki

L V Syro

E Tartour

C Tavares

M Terzolo

M Theodoropoulou

H J L M Timmers

F Tomao

T Y N Tong

D J Topliss

L Torregrossa

G Treglia

V H M Tsang

K Tsilidis

T Tsutsui

S Ulisse

I van der Ploeg

M van Hoek

D van Rooyen

D J Vander Griend

A Vega

J Verbalis

C Verroken

M Vetter

R Vigneri

E Vincent

E E Vincent

D Viola

M Vitale

L Vroonen

S G Waguespack

H Wallace

J-Y Wang

L S Ward

P J Wees

M C Wehr

V Weiss

K Welen

Y E Whang

B Whitelaw

J Wietrzyk

J Wu

Y-L Wu

B Wyse

M Yamaguchi

H Yamashita

L Yang

S-c Yeung

N Yildizbayrak

M C Zatelli

G Zaun

W Zwart

